# Stepwise Management of Status Asthmaticus Refractory to Initial Therapy: A Case Report

**DOI:** 10.7759/cureus.89652

**Published:** 2025-08-08

**Authors:** Brandon Weissman, Shafayath Chowdhury, Kevin Shen, Sumi Singh

**Affiliations:** 1 Department of Otolaryngology, Lake Erie College of Osteopathic Medicine, Elmira, USA; 2 Department of Anesthesiology and Critical Care, Lake Erie College of Osteopathic Medicine, Elmira, USA; 3 Department of Medicine, Lake Erie College of Osteopathic Medicine, Pittsburgh, USA; 4 Department of Internal Medicine, University at Buffalo, Buffalo, USA

**Keywords:** asthma, medical icu, refractory asthma, severe refractory asthma, status asthmaticus

## Abstract

Asthma is one of the most prevalent chronic respiratory illnesses, significantly impacting patients through shortness of breath and even death. Acute exacerbations are usually controlled with a short-acting beta agonist, such as an albuterol inhaler, as well as long-acting agents to prevent the occurrence of exacerbations and status asthmaticus. Status asthmaticus is an emergent episode of asthma that is refractory to standard treatment. This disease presents as tachycardia, tachypnea, and dyspnea. The forced expiratory volume measures the severity of asthma in one second and the serial peak expiratory flow rate. Proper treatment is vital for patient survival. This case report reviews the proper treatment of a patient in her mid-30s presenting to the emergency department due to an asthmatic attack refractory to albuterol. The patient went through a five-stage treatment plan. First, the patient was treated with inhaled beta-2 agonist (albuterol) and corticosteroids (prednisone, dexamethasone, and methylprednisolone). The patient did not improve with these treatments and was given the anticholinergic agent ipratropium bromide in an attempt to increase bronchodilation. Nebulized racemic epinephrine was then added to the patient to optimize maximum bronchodilation and vasoconstriction in an attempt to reduce airway edema and inflammation. To reduce ventilator peak airway pressures through sedation and paralytics, rocuronium and cisatracurium (Nimbex) were administered. Ketamine was added as a sedative and bronchodilator. Propofol and midazolam (Versed) were used to sedate the patient for mechanical ventilation. After the acute episode, maintenance therapy included inhaled corticosteroids (budesonide), a long-acting beta-2 agonist (arformoterol), a long-acting muscarinic antagonist (revefenacin), and montelukast (a leukotriene receptor antagonist). This case illustrates the importance of status asthmaticus treatment as a vital, stepwise process that focuses on bronchodilation, maintaining the airway, mechanical ventilation, sedation, and reducing inflammation and paralysis.

## Introduction

Asthma is among the most prevalent chronic respiratory illnesses, affecting around 250 million individuals [1]. The pathophysiology of asthma is related to inflammatory processes directed at total immunoglobulin E (IgE) serum levels via allergen exposure [2]. Asthma develops in the bronchial tree, where the bronchi are composed of smooth muscle and elastic fibers that contribute to the integrity of the wall. Inflammation of the bronchioles can cause bronchoconstriction and damage the relaxation efficiency of the bronchioles. IgE antibodies, released by plasma cells, bind to mast cells and basophils, triggering the release of cytokines. These mast cells release histamine, prostaglandins, and leukotrienes, which contract smooth muscle and cause airway tightening [3]. Interleukin 13 is released, which causes remodeling, fibrosis, and hyperplasia [4]. Eosinophils, basophils, neutrophils, and T cells are released into the lungs for inflammation. The remodeling makes the airway thicker, causing decreased compliance and increased breathing work [5]. This disease typically begins in childhood and is often associated with allergies [6]. Treatment is determined through guidelines by the National Asthma Education and Prevention Program and the Global Initiative for Asthma (GINA). Patients are usually given a rapid-onset bronchodilator (Albuterol) and, based on the GINA guidelines, a low-dose glucocorticoid inhaler or long-acting beta agonist. Asthma contributes to approximately 420,000 deaths annually [7]. If asthma is refractory to treatment, the problem becomes status asthmaticus.

Status asthmaticus is a typical healthcare emergency described as an emergent asthmatic episode refractory to standard treatment. Status asthmaticus will often present with hypoxia, hypercarbia, and secondary respiratory failure. Recognizing these episodes is crucial because patient outcomes are closely correlated with timely management. Patients will present tachycardic, tachypneic, and dyspneic. Asthma patients can also present with wheezing on pulmonary auscultation. Status asthmaticus can affect circulation, leading to ventricular septal deviation to the left, enlarged right ventricle, increased left ventricular afterload, and increased right ventricular afterload due to increased pulmonary arterial pressure [8]. Status asthmaticus can cause pulsus paradoxus greater than 12 mmHg during inspiration. For management, serial peak expiratory flow rates (PEFRs) and forced expiratory volumes in one second (FEV1) are used to measure disease severity. A PEFR of less than 120 L/minute and an FEV1 of less than 1 L indicate severe disease [8]. These values are also used to determine hospitalization, where 40-70% of intended responses to FEV1 and PEFR are considered inadequate and require admission [8]. During an asthma attack, the first line of treatment is an inhaled beta-2 agonist (albuterol) and oxygen management. Inhaled corticosteroids are added to reduce relapse in the next seven to ten days [9]. If a patient is deteriorating despite treatment, respiratory support is used to manage the disease. To best learn the proper treatment of status asthmaticus, we present a patient who presented to the emergency department (ED) due to an asthma exacerbation refractory to albuterol therapy.

## Case presentation

**Figure 1:**
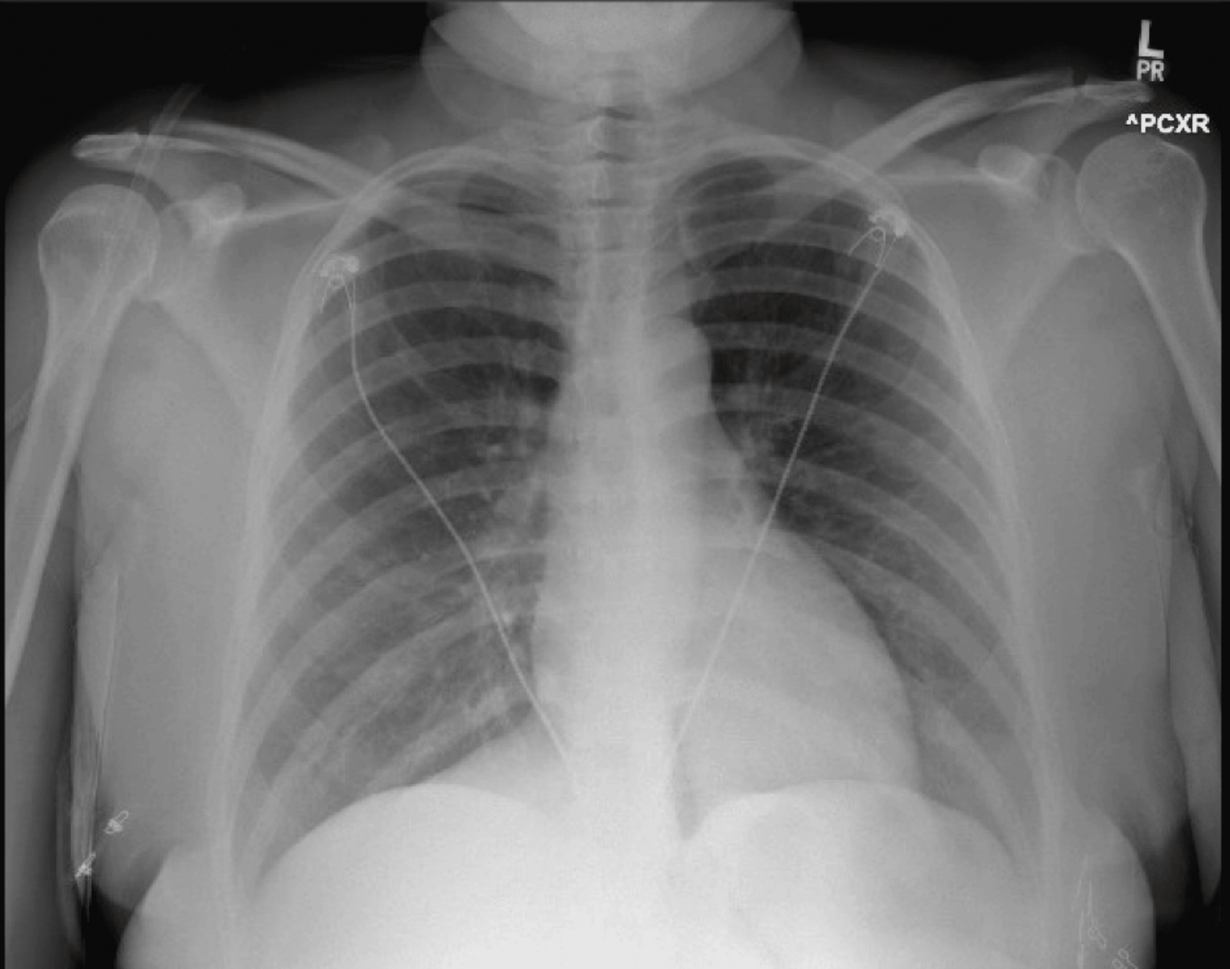
Coronal chest X-ray on presentation

A patient with a past medical history of anxiety and depression presented to the ED with a two-day history of shortness of breath, nonproductive cough, congestion, and rhinorrhea. She reported using her albuterol inhaler throughout the day with no relief and continued to feel short of breath, leading to her presentation to the ED. The patient denies any known history of asthma, childhood asthma, or reactive airway syndrome; she has a five-pack-year history of cigarette use but endorsed quitting earlier this year. In the ED, the patient had an oxygen saturation of 93% and received two breathing treatments with dexamethasone (Decadron). The patient had a normal chest X-ray with no acute pathology present, as seen in Figure 1. The patient was admitted for observation and treated with a prednisone course, ipratropium-albuterol (DuoNeb) 0.5-2.5 mg/3 mL four times per day (QID), albuterol as needed (PRN), and guaifenesin (Mucinex) QID. The day after admission, the patient's blood pressure was 152/94 mmHg, pulse 120 bpm, and respirations 28. The patient became unresponsive. A stat venous blood gas was obtained, as shown in Table 1.

**Table 1:** Arterial blood gas on presentation pH: potential of hydrogen; pCO_2_: partial pressure of carbon dioxide; pO_2_: partial pressure of oxygen

Parameter	Result	Reference range
pH	7.15	7.31-7.41
pCO_2_	82 mmHg	40-52
pO_2_	127 mmHg	30-50
O_2_ saturation	97%	95-100%
Finger stick glucose	163 mg/dL	70-99 mg/dL

The patient was promptly transferred to the intensive care unit (ICU) for acute hypercarbic respiratory failure; the patient was administered 125 mg of intravenous Solu-Medrol and racemic epinephrine via nebulization. Initial improvement was minimal, and the patient still had significant expiratory wheezes and used accessory muscles to breathe; the patient was intubated. The patient's peak pressure remained 40-50 cm H_2_O (reference range: 15-25 cm H_2_O); the patient's initial sedation was preceded by fentanyl, but the peak pressure continued to be elevated. A dose of 50 mg of rocuronium did not improve peak pressure, so a propofol drip was initiated, and the peak pressure remained elevated. A ketamine drip was added; however, peak pressure remained in the 50s. Precedex was stopped, and Versed was started. The patient required a dose of norepinephrine bitartrate (Levophed) infusion, and a central line was placed. After the procedure, the patient became bradycardic and had pulseless electrical activity (PEA), requiring chest compressions for three minutes and one dose of epinephrine. This episode of PEA was attributed to obstructed PEA. The patient was successfully resuscitated, and peak pressures remained in the 50s, measured in cm H_2_O. The patient was diagnosed with status asthmaticus and remained ventilated. Increased endotracheal yellow secretions began to form on day 3 of intubation, which were cultured, and the patient was started on a course of ceftriaxone. Throughout the hospital course, the patient's peak pressures occasionally remained elevated, reaching 57 cm H_2_O. At this point, cisatracurium (Nimbex) was administered, which brought the pressures down to less than 40 cm H_2_O. The patient was maintained on continuous ipratropium-albuterol (DuoNeb) 0.5-2.5 mg/3 mL, four times a day, a once-daily dose of montelukast 10 mg, and methylprednisolone 81.25 mg every eight hours. The patient was extubated after 16 days in the ICU. The patient was given a presumed diagnosis of asthma. The patient was put on a regimen of continuous budesonide, arformoterol twice daily, revefenacin daily, and montelukast nightly.

## Discussion

Status asthmaticus represents a serious asthma exacerbation that fails to respond to standard bronchodilators and leads to respiratory failure [10]. The treatment of status asthmaticus requires prompt medical recognition followed by forceful interventions to overcome airway obstruction and inflammation while enhancing gas exchange and preventing respiratory arrest complications.

The patient in this report underwent first-stage treatment with inhaled beta-2 agonists (albuterol) and corticosteroids (prednisone, dexamethasone, and methylprednisolone). The primary mechanism of albuterol involves activating beta-2 adrenergic receptors in bronchial smooth muscle to induce relaxation, which results in bronchodilation. The fast-acting bronchodilation effect of this medication helps ease bronchospasm and relieve patients [11]. Combining inhaled budesonide with systemic prednisone and methylprednisolone reduces inflammation by inhibiting cytokine production and decreases edema and mucus formation, thereby enhancing airway responsiveness and minimizing the occurrence of relapse [12].

The patient needed additional medications because of ongoing respiratory distress that did not improve with first-line treatments. The lack of response to peak pressures determined this. The anticholinergic agent ipratropium bromide was added to albuterol (DuoNeb) to treat airway constriction by blocking muscarinic receptors. The medication blocks vagally mediated bronchoconstriction while enhancing the bronchodilatory actions of albuterol [13].

Racemic epinephrine was introduced via nebulization when initial treatments were inadequate. Epinephrine, acting on alpha and beta-adrenergic receptors, contributes to bronchodilation and vasoconstriction, temporarily reducing airway edema and inflammation, particularly useful in acute emergency settings [14]. However, its use in asthma is typically reserved for severe exacerbations due to its cardiovascular side effects.

The patient required sedation and paralytics because peak airway pressures remained elevated despite receiving the maximum possible medical treatment. Neuromuscular blockers like rocuronium and cisatracurium (Nimbex) induced skeletal muscle paralysis, eliminating muscle tone and patient-ventilator asynchrony. This indirectly reduces peak airway pressures, facilitating ventilation in severe status asthmaticus. However, these agents do not directly enhance lung compliance but rather improve ventilation parameters by reducing muscular resistance and respiratory drive [15,16].

Ketamine was also used in the treatment regimen because it has a dual role as a sedative and bronchodilator through N-methyl-D-aspartate receptor antagonism and sympathomimetic actions, which result in bronchodilation and decreased airway resistance [17]. Propofol and midazolam (Versed) were used for sedation purposes to calm the patient and increase cooperation with mechanical ventilation, which is crucial in critically ill asthmatic patients to help maintain ventilator synchrony.

For maintenance therapy following the acute episode, budesonide (inhaled corticosteroid), arformoterol (long-acting beta-2 agonist), revefenacin (long-acting muscarinic antagonist), and montelukast (leukotriene receptor antagonist) were prescribed. Arformoterol extends bronchodilation duration by continuously stimulating beta-2 adrenergic receptors, providing sustained airway patency [18]. Revefenacin complements bronchodilation by prolonged blockade of muscarinic receptors, reducing cholinergic-mediated bronchoconstriction and mucus secretion [19]. Montelukast targets leukotriene receptors, reducing bronchial inflammation and hyperreactivity and improving asthma control by interrupting inflammatory pathways mediated by leukotrienes [20].

The management of status asthmaticus demands a multiclass pharmacological strategy that addresses bronchoconstriction, inflammation, and mucus production through the complex clinical case presented.

## Conclusions

The global health community continues to face asthma as a major concern because of its widespread occurrence and dangerous complications, including status asthmaticus. Successfully treating status asthmaticus depends on immediate medical detection followed by forceful intervention. The case illustrates the intricate process of treating refractory asthma exacerbations by showing how bronchodilators, corticosteroids, anticholinergics, neuromuscular blockers, and sedatives must be used together to treat severe symptoms in conjunction with starting mechanical ventilation. The prevention of chronic inflammation and recurrence depends heavily on maintenance therapy, which includes inhaled corticosteroids, long-acting beta-agonists, muscarinic antagonists, and leukotriene receptor antagonists. Successfully managing severe asthma requires healthcare providers to understand drug mechanisms while developing personalized treatment plans to enhance patient outcomes.
